# Selective inhibitors of cardiac ADPR cyclase as novel anti-arrhythmic compounds

**DOI:** 10.1007/s00210-012-0750-2

**Published:** 2012-04-19

**Authors:** Aimo Kannt, Kerstin Sicka, Katja Kroll, Dieter Kadereit, Heinz Gögelein

**Affiliations:** 1 Sanofi-Aventis Deutschland GmbH, Industriepark Hoechst, G877, 65926 Frankfurt am Main, Germany; 2 Sanofi-Aventis Deutschland GmbH, Industriepark Hoechst, G878, 65926 Frankfurt am Main, Germany

**Keywords:** Cyclic ADP-ribose, ADP-ribosyl cyclase, Anti-arrhythmic agents, Ventricular arrhythmias, Ca channel

## Abstract

ADP-ribosyl cyclases (ADPRCs) catalyse the conversion of nicotinamide adenine dinucleotide to cyclic adenosine diphosphoribose (cADPR) which is a second messenger involved in Ca^2+^ mobilisation from intracellular stores. Via its interaction with the ryanodine receptor Ca^2+^ channel in the heart, cADPR may exert arrhythmogenic activity. To test this hypothesis, we have studied the effect of novel cardiac ADPRC inhibitors in vitro and in vivo in models of ventricular arrhythmias. Using a high-throughput screening approach on cardiac sarcoplasmic reticulum membranes isolated from pig and rat and nicotinamide hypoxanthine dinuleotide as a surrogate substrate, we have identified potent and selective inhibitors of an intracellular, membrane-bound cardiac ADPRC that are different from the two known mammalian ADPRCs, CD38 and CD157/Bst1. We show that two structurally distinct cardiac ADPRC inhibitors, SAN2589 and SAN4825, prevent the formation of spontaneous action potentials in guinea pig papillary muscle in vitro and that compound SAN4825 is active in vivo in delaying ventricular fibrillation and cardiac arrest in a guinea pig model of Ca^2+^ overload-induced arrhythmia. Inhibition of cardiac ADPRC prevents Ca^2+^ overload-induced spontaneous depolarizations and ventricular fibrillation and may thus provide a novel therapeutic principle for the treatment of cardiac arrhythmias.

## Introduction

Cyclic adenosine diphosphoribose (cADPR) was first identified as a Ca^2+^-mobilising second messenger in sea urchin eggs derived from nicotinamide adenine dinucleotide (NAD, Clapper et al. 1987; Lee et al. 1989). cADPR-regulated Ca^2+^ release from intracellular Ca^2+^stores plays a role in a variety of physiological processes such as insulin secretion(Takasawa et al. 1993), T cell activation (Howard et al. 1993), regulation of vascular tone (Geiger et al. 2000) or cardiac excitation–contraction coupling (Rakovic et al. 1996). The mechanism by which cADPR influences intracellular Ca^2+^ mobilisation remains controversial (Venturi et al. 2010).

The enzyme catalyzing cADPR formation, ADP-ribosyl cyclase (ADPRC), was first purified from *Aplysia californica* (sea slug) ovotestis (Lee and Aarhus 1991; Hellmich and Strumwasser 1991). Based on sequence comparison, two mammalian homologs have been identified: the CD38 surface antigen, a marker of lymphocyte activation and differentiation, and CD157/BST-1 (bone marrow stromal antigen 1, States et al. 1992; Itoh et al. 1994; Lee 2000) that arose from a gene duplication event (Malavasi et al. 2006). The three enzymes have only about 30 % sequence identity but contain a set of ten cysteine residues that are strongly conserved across species.

Recently, ADPR cyclase activites with properties distinct from CD38 and CD157 have been identified in a variety of mammalian tissues, for example, in brain (Ceni et al. 2003), retinal rod outer segments (Fabiano et al. 2011), heart (Xie et al. 2005), vascular smooth muscle (de Toledo et al. 2000), skeletal muscle (Bacher et al. 2004) and kidney (Nam et al. 2006). They are located intracellularly and are, for example, inhibited by low millimolar concentrations of Zn^2+^ ions. Both cADPR and NAD concentrations were not significantly different in heart and kidney and only mildly reduced in lung and brain of CD38−/− mice compared to wild-type controls (Young et al. 2006). So far, the molecular correlates of these ADPRC activities have not been determined.

The role of cADPR for Ca^2+^ release via the cardiac ryanodine receptor (RyR2) has been extensively investigated. First evidence for an activation of RyR2 by cADPR in cardiac microsomes was provided by Meszaros et al. (1993). In intact cardiomyocytes from rats and guinea pigs, cADPR injection or photorelease led to an increase in the magnitude of Ca^2+^ transients, an augmentation of contraction and an increase in the frequency of occurrence of spontaneous Ca^2+^ sparks. All of these phenomena were prevented in the presence of competitive antagonists of cADPR-induced Ca^2+^ mobilisation, 8-amino-cADPR or 8-bromo-cADPR (Rakovic et al. 1996; Iino et al. 1997; Cui et al. 1999).

Additionally, it was shown that cADPR is a mediator of the sustained phase of the angiotensin II-induced rise in intracellular Ca^2+^ and angiotensin II-stimulated hypertrophy of rat cardiomyocytes (Gul et al. 2008) and that 4,4′-dihydroxyazobenzene, an inhibitor of cellular cADPR formation, can block angiotensin II-induced cardiac hypertrophy in vivo in a two-kidney one-clip rat model (Gul et al. 2009).

In intact guinea pig cardiomyocytes, under Ca^2+^ overload through high concentrations of the beta-adrenoreceptor agonist isoproterenol or the Na^+^/K^+^-ATPase inhibitor ouabain, spontaneous generation of action potentials and Ca^2+^ waves was suppressed in the presence of 8-amino-cADPR. Moreover, cADPR infusion was associated with spontaneous electrical and contractile activity, pointing towards the possibility that cADPR may exert arrhythmogenic activity in the heart (Rakovic et al. 1999).

Here, we show that a potent and specific inhibitor of cardiac ADPR cyclase, a protein that is distinct from CD38 or the archetypical ADPR cyclase from *A. californica*, can suppress Ca^2+^ overload-induced arrhythmic events both in vitro and in vivo. Thus, cardiac ADPR cyclase inhibitors may have a therapeutic potential as anti-arrhythmic agents in, e.g., catecholaminergic polymorphic ventricular tachycardia (CPVT) or congestive heart failure.

## Methods

### Purification of recombinant human CD38 and *A. californica* ADPR cyclase

The full-length cDNA of human CD38 and ADPR cyclase from *A. californica* were used to clone expression constructs. In ORF coding for the extracellular domain of human CD38 Arg45-Ile300 (P28907), *A. californica* ADPRC Ile25-Ala282 (P29241) was cloned in frame with an insect prepromelitin signal sequence and a 6xHis tag at the C-terminal end of the coding proteins and ligated into the multiple cloning site of the baculovirus transfer vector (Kitts and Possee 1993) pVL1393 vector (AB Vector). After cotransfection of plasmids with baculovirus DNA (flashBAC gold, Oxford Expression Technologies), virus was amplificated in two steps in *Spodoptera frugiperda* cell line SF9 (Vaughn et al. 1977) in SF900II medium (Invitrogen) supplemented with 5 % fetal calf serum (FCS). The recombinant virus was harvested 5 days post-transfection. The virus titres were determined by plaque assay method (Brown and Faulkner 1977) and reached about 1 × 10^8^ pfu/ml.

*A. californica* ADPRC was expressed for 72 h post infection at a multiplicity of infection (MOI) of 3 in 1.4 × 10^6^ cells/ml in High Five cells growing in ExCell405 medium at a 1-l scale using vented 3,000-ml flasks (Corning) at 100 rpm. Human CD38 extracellular domain was expressed for 72 h post infection at 1 MOI in 1.6 × 10^6^ cells/ml in SF9 cells cultivated in SF900II medium without FCS in 1-l scale in vented 3,000-ml flasks (Corning) at 100 rpm at 27°C.

All following purification steps were carried out at 4°C using an ÄKTA explorer system (GE) for chromatography steps: His-tagged proteins from the clarified insect cell supernatant were bound directly to a metal-chelating ligand (Hochuli et al. 1988) using a 1-ml HiTrap columns HP (GE) with a flow rate of 1 ml/min. After washing with 20 CV of buffer consisting 300 mM NaCl and 50 mM Tris/HCl pH 7.5, bound protein was eluted within a linear gradient from 0–100 % buffer which is composed of 300 mM NaCl, 50 mM Tris/HCl pH 7.5 and 500 mM imidazole. Fractions were collected, analysed on SDS-PAGE, pooled and dialyzed overnight against 50 mM Tris/HCl pH 7.5. The sample was filtrated (0.22 μm, Sartorius) to separate the precipitated protein from contamination, and protein was applied on a cation exchanger, a HiTrap S HP (GE) 1-ml column. Bound protein was eluted within a linear gradient from 0 mM NaCl up to 1 M NaCl in 50 mM Tris/HCl pH 7.5. After analysis of eluted fractions, the protein solution had a purity of about 80 % and was further enriched by size exclusion chromatography on Superdex 16/60 200 HR (GE) using a buffer composed of 300 mM NaCl and 50 mM Tris/HCl pH 7.5.

### Animal investigations

All investigations with animals conform to the Guide for the Care and Use of Laboratory Animal published by the US National Institute of Health (NIH publication no. 85–23, revised 1996) and were performed by technicians specifically trained and experienced in animal care following approval by the Ethical Review Board of the State of Hessen and in accordance with the German animal protection law (application reference HMR-4A/Anzeige47).

### Preparation of cardiac sarcoplasmic reticulum membranes

Sarcoplasmic reticulum membranes from pig and rat heart were prepared according to the following protocol (Kranias et al. 1982): Pigs were anaesthetized with a single bolus of 16 mg/kg pentobarbital and then euthanised by a single bolus injection of 10 ml saturated KCl. Immediately thereafter, the thorax was opened, and the heart was removed and put into ice-cold medium I (30 mM Tris–Malat, pH 7.0, 0.3 M sucrose, 5 mg/ml leupeptin, 0.1 mM PMSF). Rats were anaesthetized by 3.5 % (*v*/*v*) isoflurane inhalation (vaporizer flow rate 0.6–0.8 l/min) and sacrificed by cervical dislocation. Hearts were removed immediately and put into ice-cold medium I. The left ventricles of freshly explanted hearts were cleaned of fat and connective tissue and cut into small pieces. The pieces were briefly rinsed with ice-cold medium I and homogenised in ice-cold medium I (5 ml/g tissue) using a Waring blender (for pig heart) or an Ultra-Turrax device (for rat heart). The homogenate was then centrifuged for 10 min at 5,500 × g. The supernatant was passed through four layers of Miracloth (Merck Biosciences, Darmstadt, Germany) and centrifuged at 12,000 × g for 25 min. The supernatant was again filtered through four layers of Miracloth and then centrifuged at 143,000 × g for 60 min. The resulting pellet was re-suspended in medium II (20 mM Tris–Malat, pH 7.0, 0.6 M KCl, 0.3 M sucrose, 5 mg/ml leupeptin, 0.1 mM PMSF) using an Ultra-Turrax, and the suspension was again centrifuged at 143,000 × g for 60 min. The pellet was re-suspended in medium I and again centrifuged at 143,000 × g for 60 min. The pellet from this last centrifugation step was re-suspended in medium III (20 mM Tris–Malat, pH 7.0, 0.1 M KCl, 0.3 M sucrose, complete protease inhibitors (Roche, Penzberg, Germany) without EDTA) to give a final protein concentration between 5 and 10 mg/ml. The suspension was aliquoted, shock-frozen in liquid nitrogen and stored at −80°C.

### Measurement of nucleoside diphosphoribosyl cyclase activity

Nicotinamide hypoxanthine dinucleotide (NHD) was taken as a surrogate for NAD to measure ADPR cyclase activity as described by Graeff et al. (1996). In the reaction catalysed by the cyclase, NHD is converted to cyclic inosine diphosphoribose (cIDPR) that can be detected via its fluorescence in the visible spectrum. The following protocol was used to identify compounds inhibiting the cyclase activity: Test compounds (stock 3 mM in DMSO) were diluted to 30 μM in reaction buffer (20 mM Tris–Malat, pH 7.0, 0.1 M KCl, 0.3 M sucrose, complete protease inhibitors without EDTA). In a 384-well small volume microtitre plate (Greiner Bio-One, Frickenhausen, Germany), 2 μl of test compounds was mixed with 2 μl of cardiac sarcoplasmic reticulum (SR) membranes diluted in the same buffer and incubated for 30 min at room temperature, and the reaction was started by the addition of 2 μl of 750 μM NHD in the same buffer. The fluorescence of the reaction mixture (excitation wavelength 275 nm, emission wavelength 410 nm) was measured directly after the addition of substrate (*t*_0_) and after several incubation periods (e.g., 30, 60, 90 min). Between measurements, the plate was incubated at 37°C. The cyclase activity of each reaction mixture was then defined by the change in fluorescence with time, i.e., the slope of the line in the fluorescence versus time plot obtained by linear regression.

### In-gel activity assay

An in-gel activity assay was performed essentially as described by Xie et al. (2005), with slight modifications: Rat cardiac SR preparations were separated on a non-reducing SDS-PAGE gel (4–12 %). For renaturation of ADPRC, the gel was incubated for 30 min in 50 mM Tris–HCl, pH 7.4, and 0.3 % Triton X-100 at room temperature and then transferred to a buffer composed of 50 mM Tris–HCl, pH 7.4, and 0.1 % Triton X-100. After 10-min incubation at ambient temperature, the gel was incubated for 60 min at room temperature in the same buffer additionally containing 250 μM NHD, and ADPRC was visualised as a fluorescent band with a UV lamp (254 nm).

### Determination of anti-arrhythmic properties of ADPR cyclase inhibitors in vitro

Anti-arrhythmic properties of cardiac ADPR cyclase inhibitors identified with the assay described above were probed for by determining their ability to prevent the formation of spontaneous action potentials in guinea pig papillary muscle cells following high-frequency electrical stimulation. The experimental protocol was as follows: Guinea pigs (Dunkin Hardley Pirbright), weighing approximately 400 g, were sacrificed by concussion followed by exsanguination. Immediately after opening of the thorax, the heart was removed and immersed in Tyrode solution (in millimole per litre): 136 NaCl, 3.3 KCl, 1.2 KH_2_PO_4_, 1.1 MgSO_4_, 2.5 CaCl_2_, 5 Glucose, 10 HEPES, pH 7.4, gassed with oxygen), at room temperature. The left papillary muscle was removed and mounted in a measuring chamber, containing Tyrode solution heated to 37°C. The time from removal of the heart until mounting the papillary muscle in the chamber was approximately 3 min.

Immediately after mounting of the papillary muscle, it was stimulated with rectangular pulses of 1 to 4 V and a duration of 1 to 3 ms, at a frequency of 1 Hz, by means of a computer programme (MFK, Niedernhausen, Germany). The bath was continuously perfused with Tyrode solution using a roller pump (TL, Meredos GmbH, Bovenden, Germany) at a rate of 4 ml/min. The solution was pre-heated to 37°C. Action potentials (APs) were recorded with a glass microelectrode, filled with 3 M KCl solution. The electrodes were fabricated from borosilicate glass (item number: 1B150F-4, World Precision Instruments, Sarasota, USA), by means of a microelectrode puller (DMZ-Universal Puller, Zeitz Instruments, Martinsried, Germany). The electrodes had an electrical resistance of 5 to 10 megaohms. The electrical signal was recorded with an amplifier (Model 309, Harvard Apparatus GmbH, March-Hugstetten, Germany), and stored in a computer system.

After an equilibration period of 30 min, an AP was recorded, and the test substance (or vehicle) was added to the perfusion solution. Fifteen minutes later, an AP was recorded, and the perfusion solution was modified: KCl and KH_2_PO_4_ were omitted, and CaCl_2_ was increased to 5.5 mM. After an additional 15 min, an AP was recorded, and the tissue was paced at 4 Hz for 30 s. Then, pacing was stopped, and frequently, spontaneous APs were observed. The number of spontaneous APs occurring within 6 s after cessation of pacing was counted. In the presence of vehicle, the number of spontaneous APs in the 6-s period was approximately 13. For each compound tested, ten experiments each for compound and vehicle control were performed. The number of spontaneous APs in each group was compared by means of the Student's *t* test.

### Determination of anti-arrhythmic properties of ADPR cyclase inhibitors in vivo

Anti-arrhythmic properties of ADPR cyclase inhibitors were measured in vivo in anaesthetized guinea pigs. For this purpose, guinea pigs were anaesthetized with pentobarbital (100 mg/kg i.p.) and ventilated with 40 % (*v*/*v*) oxygen (O_2_). Adequacy of anaesthesia was followed by monitoring of the vital signs and the palpebral and foot retraction reflexes. After 30 min, the test compound was applied at 3 mg/kg as a bolus injection. Five minutes later, ouabain was infused at a rate of 30 μg/kg per min, causing a strong continuous increase in contractility. In the presence of the background sample (bolus injection of vehicle only), ventricular fibrillation was observed approximately 12 min after infusion of ouabain, and cardiac arrest occurred after approximately 15 min.

## Results

### Cardiac ADPR cyclase is distinct from CD38 

**Fig. 1:**
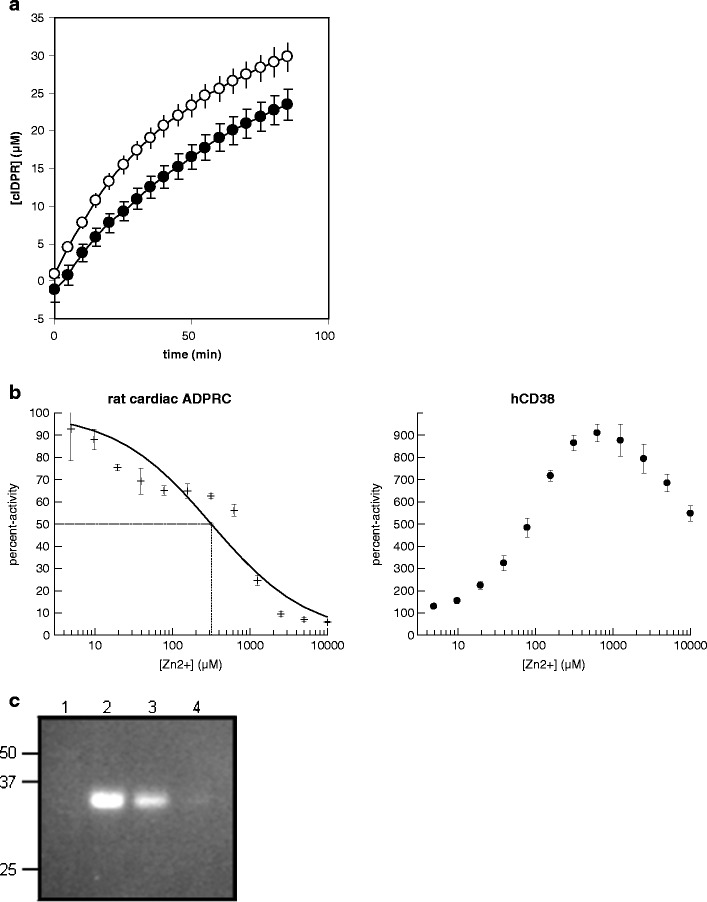
**a** Formation of cIDPR by purified cardiac sarcoplasmic reticulum vesicles. *Filled circles*, rat cardiac SR; *open circles*, pig cardiac SR (*n* = 16 each). Specific IDPR cyclase activity was estimated as 6.5 nmol min^−1^ mg protein^−1^ for rat and 0.63 nmol min^−1^ mg protein^−1^ for pig SR. **b** Effect of Zn^2+^ ions on the activity of rat cardiac SR ADPR cyclase, CD38 and *A. californica* ADPR cyclase. For the rat cardiac ADPR cyclase, the *dashed line* denotes the IC_50_ value that was determined as 320 ± 55 μM (*n* = 3). **c** In-gel activity assay of partially purified ADPR cyclase from rat cardiac SR vesicles. Activities in aqueous solutions of the samples in *lanes 2*, *3* and *4* corresponded to 21, 15 and 4 RFU/s, respectively

Sarcoplasmic reticulum vesicles prepared from rat or pig hearts were found to contain ADPR cyclase activity measured using NHD as a surrogate substrate (Fig. 1a), as previously described by Meszaros et al. (1997) and Xie et al. (2005). Unlike CD38, the cardiac SR enzyme was determined to be inhibited by Zn^2+^ (Fig. 1b) showing that the cardiac protein is different from the known mammalian ADPR cyclases, CD38 and Bst1/CD157, that are both activated by Zn^2+^ (Hirata et al. 1994; Xie et al. 2005). In an in-gel activity assay with partially purified rat heart ADPR cyclase, a single fluorescent band corresponding to an apparent molecular weight of approximately 35 kDa was observed (Fig. 1c), again in line with previous observations (Xie et al. 2005).

### Identification of inhibitors of cardiac ADPR cyclase

**Fig. 2:**
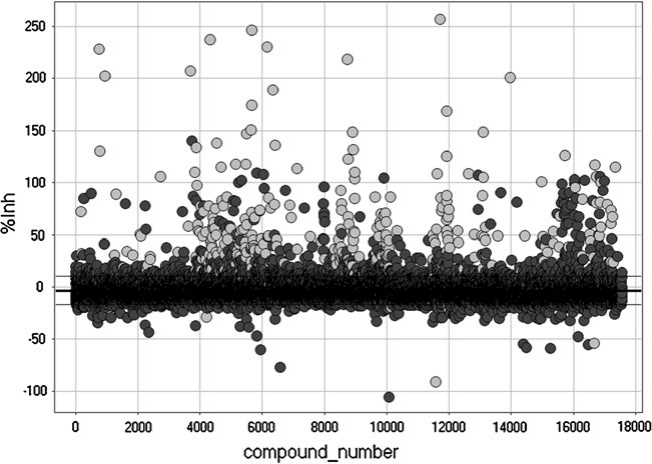
High-throughput screen for small-molecule inhibitors of cardiac ADPR cyclase. 17,567 compounds were tested at 10 μM for inhibition of pig cardiac ADPR cyclase. Autofluorescent compounds with fluorescence at *t* = 0 are shown in *light grey* and were removed before further analysis (327 compounds). *Horizontal black lines* denote the mean percent inhibition value over all compounds ± standard deviation that was determined as −3.2 ± 13.8. Non-fluorescent compounds with a percent inhibition above mean + 3 SD were considered as screening hits (93 compounds)

**Fig. 3:**
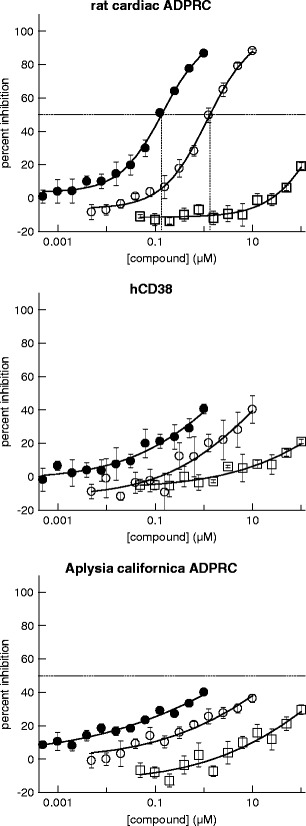
Compounds SAN4825 and SAN2589 are selective inhibitors of cardiac ADPR cyclase. Inhibition of ADPRC activity of cardiac ADPRC (*upper panel*), CD38 (*middle panel*) and *A. californica* ADPRC are shown for 4825 (*filled circles*), 2589 (*open circles*) and DHAB (*open squares*). IC_50_ values for SAN4825 and SAN2589 on cardiac ADPRC were determined as 0.13 ± 0.02 and 1.3 ± 0.2 μM, respectively (*n* = 3 each)

A diverse collection of 17,567 small-molecule compounds was screened at 10-μM concentration for inhibitors of pig heart ADPR cyclase (Fig. 2). All compounds without autofluorescence observed at *t* = 0 that gave >38.2 % inhibition (mean percent inhibition plus three standard deviations) were considered as screening hits and re-tested over the concentration range between 0.3 and 40 μM; 71 compounds with a concentration response and IC_50_ values below 40 μM could be identified. After clustering and testing of compounds with related structures, two structural series could be identified with activities of their most potent compounds being in the single-digit nanomolar range. This is several orders of magnitude more potent than the only previously described inhibitor of a non-CD38/CD157 mammalian ADPR cyclase, dihydroxyazobenzene, which was found to have an IC_50_ value of >100 μM (Fig. 3), which is in good agreement with published data (Nam et al. 2006). Further details and structure–activity relationships for the two series will be described elsewhere.

**Fig. 4:**
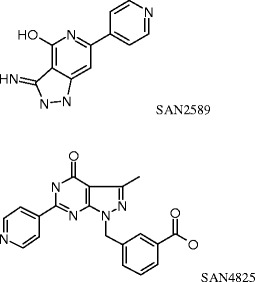
Structures of compound SAN2589 and SAN4825, identified as potent and selective inhibitors of cardiac ADPR cyclase

Compounds from both series were found to be specific for the cardiac ADPR cyclase as compared to CD38 and *A. californica* ADPR cyclase, again pointing towards the cardiac protein being structurally and/or mechanistically distinct from the known ADPR cyclases (Fig. 3). In addition, two representative compounds, SAN2589 and SAN4825 (for structures, see Fig. 4), were tested for their selectivity against 33 other proteins. With the exception of the adenosine A_2A_ receptor where ligand binding was inhibited by 81 % in the presence of SAN4825, no binding to any other tested protein was inhibited by more than 50 % by any of the two tested compounds tested at 10 μM (Table 1).

**Table 1:** Selectivity of SAN4825 and SAN2589 against a panel of 33 proteins

Assay	SAN2589	SAN4825
A1 (h) (antagonist radioligand)	83 ± 3	72 ± 1
A2A (h) (agonist radioligand)	61 ± 6	**19** **±** 1
Alpha 1A (antagonist radioligand)	98 ± 7	94 ± 6
Alpha 2A (h) (antagonist radioligand)	92 ± 12	108 ± 5
Beta 1 (h) (agonist radioligand)	95 ± 1	93 ± 5
Beta 2 (h) (agonist radioligand)	104 ± 8	95 ± 12
CB1 (h) (agonist radioligand)	89 ± 24	89 ± 6
CB2 (h) (agonist radioligand)	101 ± 4	93 ± 1
D1 (h) (antagonist radioligand)	120 ± 1	116 ± 4
D2S (h) (antagonist radioligand)	107 ± 15	89 ± 4
Glycine (strychnine-sensitive) (antagonist radioligand)	109 ± 13	80 ± 6
H1 (h) (antagonist radioligand)	105 ± 10	98 ± 5
H2 (h) (antagonist radioligand)	119 ± 19	90 ± 4
M1 (h) (antagonist radioligand)	102 ± 3	88 ± 3
M2 (h) (antagonist radioligand)	114 ± 2	86 ± 8
M3 (h) (antagonist radioligand)	97 ± 0.4	81 ± 2
N neuronal alpha 4beta 2 (h) (agonist radioligand)	114 ± 10	93 ± 6
N muscle-type (h) (antagonist radioligand)	99 ± 6	96 ± 8
Mu (MOP) (h) (agonist radioligand)	104 ± 6	96 ± 16
PCP (antagonist radioligand)	103 ± 1	117 ± 8
5-HT1A (h) (agonist radioligand)	97 ± 8	107 ± 10
5-HT2A (h) (antagonist radioligand)	123 ± 14	98 ± 5
5-HT2B (h) (antagonist radioligand)	103 ± 5	100 ±9
Ca^2+^ channel (L, dihydropyridine site) (antagonist radioligand)	118 ± 29	109 ± 0.2
KV channel (antagonist radioligand)	131 ± 6	93 ± 9
SKCa channel (antagonist radioligand)	88 ± 3	96 ± 0.4
Cl^−^ channel (GABA-gated) (antagonist radioligand)	97 ± 1	89 ± 0
Norepinephrine transporter (h) (antagonist radioligand)	95 ± 1	102 ± 0.3
dopamine transporter (h) (antagonist radioligand)	103 ± 29	94 ± 5
PDE3A (h)	96 ± 2	90 ± 1
Acetylcholinesterase (h)	107 ± 2	89 ± 5
MAO-A (h)	98 ± 11	98 ± 3
ATPase (Na^+^/K^+^)	99 ± 1	103 ± 2

Compounds were tested in duplicates at 10 μM. Results shown are expressed as percent activity of control ± standard deviation

### Antiarrhythmic activity of cardiac ADPR cyclase inhibitors

**Fig. 5:**
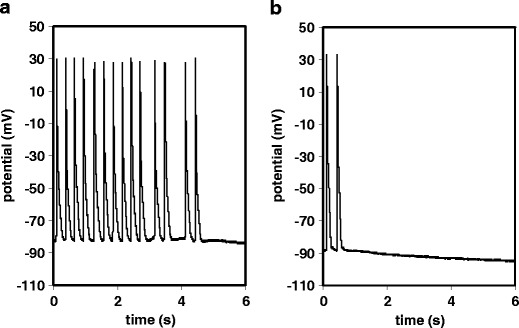
Representative experimental traces showing the generation of spontaneous action potentials in guinea pig papillary muscle after high-frequency pacing. At *t* = 0, electrical stimulation was switched off. **a** Vehicle control and **b** 3 μM SAN4825

**Fig. 6:**
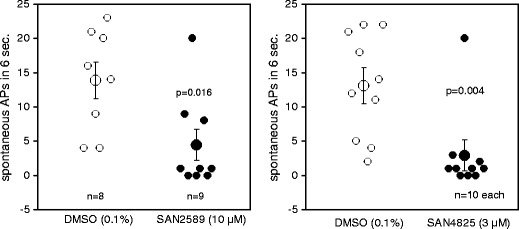
The ADPR cyclase inhibitors SAN2589 and SAN4825 significantly reduced the number of spontaneous action potentials observed within 6 s after high-frequency pacing of guinea pig papillary muscle. *The given values of n* refer to the number of independent experiments (one papillary muscle isolated per animal)

Out of the potent and selective inhibitors of cardiac ADPR cyclase identified in the experiments described above, the two structurally distinct compounds SAN4825 and SAN2589 were selected for further characterisation in models of ventricular arrhythmia. First, both compounds were tested for their ability to prevent the occurrence of spontaneous action potentials in guinea pig papillary muscle cells following high-frequency electrical stimulation. Representative traces are shown in Fig. 5; the results of the study are summarised in Fig. 6. At 3 μM, SAN4825 significantly reduced the number of delayed after depolarisations within 6 s following rapid pacing from 13.1 ± 2.7 to 2.9 ± 1.9 (*n* = 10 each, *p* = 0.004). With vehicle only, the number of spontaneous action potentials was equal to or above 11 in seven out of ten experiments whereas addition of SAN4825 reduced the number of spontaneous electrical activities to equal to or below three in nine out of ten experiments. There was no difference in membrane potential (−81.5 ± 5.3 mV for vehicle versus −77.6 ± 10.2 mV for SAN4825)

This anti-arrhythmic effect was confirmed with the structurally unrelated ADPR cyclase inhibitor, SAN2589 that, at 10-μM concentration, significantly reduced the number of spontaneous action potentials after high frequency stimulation, from 13.9 ± 2.7 to 4.4 ± 2.3 (*n* = 8 and 9, respectively, *p* = 0.016). Again, no significant differences in membrane potential were observed (−86.5 ± 5.0 mV for vehicle versus −81.4 ± 9.2 mV for SAN2589).

**Fig. 7:**
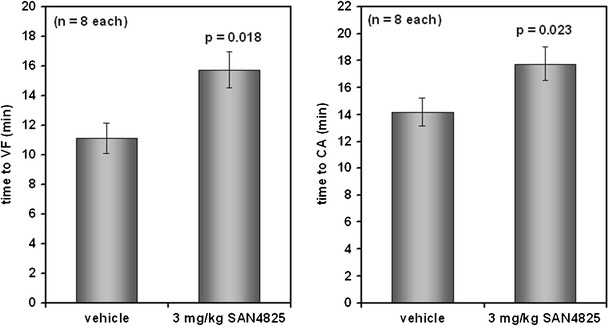
Compound SAN4825, at 3 mg/kg i.v., significantly prolongs the time to ventrular fibrillation (VF) and cardiac arrest (CA) upon infusion of ouabain at 30 μg/kg/min

Subsequently, compound SAN4825 was tested in an in vivo model of Ca^2+^ overload-induced ventricular arrhythmia (Fig. 7). Five minutes after an i.v. bolus injection of 4825 or vehicle at 3 mg/kg, the Na^+^/K^+^ pump inhibitor ouabain was infused at a rate of 30 μg/kg per min, leading to a pronounced Ca^2+^ overload and the development of ventricular fibrillation and cardiac arrest within approximately 11 and 14 min, respectively. In this setting, compound SAN4825 was determined to significantly prolong the time between ouabain infusion and resulting ventricular fibrillation (from 11.1 ± 1.0 to 15.7 ± 1.2 min, *n* = 8 each, *p* = 0.018) and cardiac arrest (from 14.2 ± 0.9 to 17.8 ± 0.9 min, *n* = 8 each, *p* = 0.023).

## Discussion

We have isolated sarcoplasmic reticulum vesicles from pig and rat heart with ADPR cyclase activity as measured using the surrogate substrate NHD. Inhibition by sub-millimolar concentrations of Zn^2+^ clearly showed that the enzyme is different from the two known mammalian ADPR cyclases, CD38 and CD157, that are both activated by Zn^2+^ (Hirata et al. 1994; Xie et al. 2005). The presence of such an activity has been described in earlier reports for dog and rat sarcoplasmic reticulum (Xie et al. 2005; Meszaros et al. 1997). Material from pig heart was used to screen for inhibitors of ADPR cyclases, and compounds with nanomolar affinities to both pig and rat cardiac ADPR cyclases were identified. In isolated guinea pig papillary muscle, two compounds from distinct structural classes were found to significantly inhibit the generation of pacing-induced spontaneous APs that are most likely the result of delayed after depolarisations (Hoffman and Rosen 1981; Hiraoka 1987). In an in vivo model of Ca^2+^ overload-induced ventricular arrhythmia, the ADPR cyclase inhibitor SAN4825 significantly prolonged the time to ventricular fibrillation and cardiac arrest upon infusion of the glycoside ouabain.

To our knowledge, this is the first description of potent and selective inhibitors of cardiac ADPR cyclase and their effectiveness as anti-arrhythmic agents. To date, the only known small-molecule compounds inhibiting a non-CD38/CD157-like ADPR cyclase are 2,2′-dihydroxyazobenzene (DAB) and 4,4′-dihydroxyazobenzene (DHAB) which was first identified as an inhibitor of kidney ADPR cyclase (Nam et al. 2006; Kim et al. 2008) and later also described as an inhibitor of cardiac ADPR cyclase (Gul et al. 2009). However, in agreement with our results, DHAB was determined to be an only moderate inhibitor of the partially purified ADPR cyclase, with an IC_50_ value of slightly greater than 100 μM (Nam et al. 2006). Interestingly, the cellular activity of DHAB as an inhibitor of angiotensin-II that induced an increase in cADPR and Ca^2+^ release in both renal mesangial cells (Nam et al. 2006) and cardiomyocytes (Kim et al. 2008) was much more pronounced—in the nanomolar to low micromolar range—indicating that the cellular activity of the compound may not be conferred by direct interaction with ADPR cyclase.

Recently, in a set of very elegant studies, Kim and colleagues showed that normalization of an angiotensin-II-dependent increase in cADPR in cardiomyocytes prevents the sustained rise in [Ca^2+^]_i_ and cellular hypertrophy. In addition, they could demonstrate that the in vitro effect was translated into an in vivo amelioration of cardiac hypertrophy and an improvement in cardiac function upon i.p. administration of DAB (1.5 mg/kg/day) in a rat two-kidney-one-clip (2K1C) model (Kim et al. 2008). In our study reported here, we show that both in vitro and in vivo, direct inhibition of ADPR cyclase also has an anti-arrhythmic effect in models of cellular or cardiac Ca^2+^ overload.

Up to 50 % of mortality in heart failure does not result from pump failure but from ventricular tachyarrhythmias (Grimm and Maisch 2002) that are the consequence of dysfunctions in cardiac Ca^2+^ homeostasis: Resting (diastolic) Ca^2+^ which is increased in heart failure(Gwathmey et al. 1990) as well as SR Ca^2+^ leak in a rare genetic disease called CPVT (Lehnart et al. 2008) can result in transient inward currents mediated by the sarcolemmal Na^+^/Ca^2+^ exchanger that, in turn, can lead to DADs and triggered activity. Both HF and CPVT have been linked to aberrant Ca^2+^ release through ‘leaky’ RyR2 channels (see Lehnart et al. 2009; Cerrone et al. 2009; Kushnir and Marks 2010 for recent reviews).

Injection of cADPR at concentrations between 100 nM and 3 μM into rat ventricular myocytes was reported to lead to a sustained rise in resting Ca^2+^and an increase in the frequency of spontaneous Ca^2+^ waves, while the cADPR receptor antagonist, 8-amino-cADPR, could reduce number and amplitude of spontaneous Ca^2+^ waves (Prakash et al. 2000). In ventricular myocytes isolated from guinea pig heart, the cADPR antagonist 8-amino-cADPR suppressed the formation of isoproterenol-provoked off-trigger action potentials and Ca^2+^ waves and ouabain-induced transient inward currents whereas cADPR infusion led to generation of spontaneous action potentials in cardiomyocytes exposed to ouabain (Rakovic et al. 1999). Other studies in intact cardiomyocytes have provided additional evidence that cADPR injection or photorelease increases the magnitude of Ca^2+^ transients, augments contraction and increases the frequency of occurrence of spontaneous Ca^2+^ sparks with these effects being prevented in the presence of 8-amino-cADPR or 8-bromo-cADPR (Rakovic et al. 1996; Iino et al. 1997; Cui et al. 1999). In contrast, Guo et al. (1996) observed no effect of cADPR or 8-amino-cADPR on Ca^2+^ transients in rat ventricular myocytes (Guo et al. 1996)

Currently, there is only scarce evidence for a potential regulation of cardiac ADPR cyclase activity or cADPR levels in disease. On the cellular level, angiotensin-II and isoproterenol have been shown to stimulate ADPRC activity and raise intracellular cADPR (Rakovic and Terrar 2000; Higashida et al. 2000; Xie et al. 2005; Gul et al. 2008). On the isolated organ level, an augmentation of cardiac cADPR was observed in Langendorff-perfused isolated rabbit hearts upon ischemia–reperfusion (Xie et al. 2003). In vivo, as mentioned above, Gul et al. (2009) demonstrated a pronounced increase in cardiac ADPR cyclase activity and cADPR levels in a rat 2K1C model that is characterised by increased angiotensin-II levels, hypertension and cardiac hypertrophy. Interestingly, 2K1C rats display an increased propensity towards ouabain-induced ventricular arrhythmia (Capasso et al. 1986) which was shown to be improved in our study here by administration of inhibitors of cardiac ADPR cyclase.

Although cyclic ADP-ribose is well established as an effective modulator of intracellular Ca^2+^ in many cell types, the mechanism by which cADPR regulates Ca^2+^ release remains unclear and is being discussed controversially (see, e.g., Venturi et al. 2010). A direct activation of RyR2 by cADPR has been proposed on the grounds of single-channel studies in planar lipid bilayers (Meszaros et al. 1993; Sitsapesan et al. 1994). However, activation of Ca^2+^ release by cADPR was reduced in the presence of ATP suggesting that under physiological conditions, at millimolar ATP concentrations, cADPR has no activating effect (Sitsapesan et al. 1994). In addition, other groups have reported that cADPR does not alter the open probability of RyR2 in planar lipid bilayers (Fruen et al. 1994; Copello et al. 2001) or that activation by cADPR is lost in the absence of FK-506 binding protein 12.6 (FKBP12.6, Tang et al. 2002) The latter effect was recently also described in intact mouse cardiomyocytes, where the increase in Ca^2+^ spark frequency upon cADPR injection was not observed in cardiomyocytes derived from FKBP12.6-knockout animals (Zhang et al. 2009). In the same study, it was also demonstrated that cADPR could displace FKBP12.6 from cardiac SR vesicles. Thus, another theory regarding the mode of action of cADPR is that of an indirect interaction with RyR2 via binding to FKBP12.6. However, other reports indicate that FKBP12.6 itself may have no influence on RyR2 opening (Barg et al. 1997; Stewart et al. 2008; Xiao et al. 2007). In addition, a role of cADPR in the regulation of the activity of sarcoplasmic reticulum Ca^2+^ ATPase has been discussed (Lukyanenko et al. 2001): In the presence of cADPR, there was an increase in the rate of Ca^2+^ uptake into cardiac SR vesicles, an elevated SR Ca^2+^ content in permeabilized ventricular myocytes and, as a consequence of this rise in luminal Ca^2+^, an increase in the frequency of Ca^2+^ sparks that was inhibited by 8-Br-cADPR. This illustrates that the mechanism of action of cADPR on SR Ca^2+^ release is complex and is still incompletely understood. It is a limitation of our study that we could not investigate the effects of our compounds on key Ca^2+^-handling proteins apart from ADPR cyclase—the molecular correlate of which is unknown—and the proteins summarised in Table 1. Nevertheless, their potency in inhibiting ADPR cyclase activity and selectivity versus a broad panel of diverse targets suggest that the two compounds could be valuable tools to further elucidate the mechanism of cADPR-induced Ca^2+^ release both in vitro and in vivo.

In summary, we have identified potent and selective inhibitors of mammalian cardiac ADPR cyclase that are distinct from the known mammalian ADPR cyclases CD38 and Bst1. Cardiac ADPR cyclase inhibitors from two different structural classes were able to suppress pacing-induced delayed after depolarisations in guinea pig cardiomyocytes. In vivo, compound SAN4825 significantly delayed ventricular fibrillation and cardiac arrest after ouabain infusion in guinea pigs. Cardiac ADPR cyclase inhibitors may thus provide a novel therapeutic principle for the prevention of ventricular arrhythmias, e.g., in conditions of catecholaminergic ventricular tachycardia or congestive heart failure.

## References

[CR1] Bacher I, Zidar A, Kratzel M, Hohenegger M (2004). Channelling of substrate promiscuity of the skeletal-muscle ADP-ribosyl cyclase isoform. Biochem J.

[CR2] Barg S, Copello JA, Fleischer S (1997). Different interactions of cardiac and skeletal muscle ryanodine receptors with FK-506 binding protein isoforms. Am J Physiol.

[CR3] Brown M, Faulkner P (1977). A plaque assay for nuclear polyhedrosis viruses using a solid overlay. J Gen Virol.

[CR4] Capasso JM, Tepper D, Reichman P, Sonnenblick EH (1986). Renal hypertensive hypertrophy in the rat: a substrate for arrhythmogenicity. Basic Res Cardiol.

[CR5] Ceni C, Müller-Steffner H, Lund F, Pochon N, Schweitzer A, De Waard M (2003). Evidence for an intracellular ADP-ribosyl cyclase/NAD^+^-glycohydrolase in brain from CD38-deficient mice. J Biol Chem.

[CR6] Cerrone M, Napolitano C, Priori SG (2009). Catecholaminergic polymorphic ventricular tachycardia: a paradigm to understand mechanisms of arrhythmias associated to impaired Ca^2+^ regulation. Hear Rhythm.

[CR7] Clapper DL, Walseth TF, Dargie PJ, Lee HC (1987). Pyridine nucleotide metabolites stimulate calcium release from sea urchin egg microsomes desensitized to inositol trisphosphate. J Biol Chem.

[CR8] Copello JA, Qi Y, Jeyakumar LH, Ogunbunmi E, Fleischer S (2001). Lack of effect of cADP-ribose and NAADP on the activity of skeletal muscle and heart ryanodine receptors. Cell Calcium.

[CR9] Cui Y, Galione A, Terrar DA (1999). Effects of photoreleased cADP-ribose on calcium transients and calcium sparks in myocytes isolated from guinea-pig and rat ventricle. Biochem J.

[CR10] De Toledo FG, Cheng J, Liang M, Chini EN, Dousa TP (2000). ADP-ribosyl cyclase in rat vascular smooth muscle cells: properties and regulation. Circ Res.

[CR11] Fabiano A, Panfoli I, Calzia D, Bruschi M, Ravera S, Bachi A (2011). Catalytic properties of the retinal rod outer segment disk ADP-ribosyl cyclase. Vis Neurosci.

[CR12] Fruen BR, Mickelson JR, Shomer NH, Velez P, Louis CF (1994). Cyclic ADP-ribose does not affect cardiac or skeletal muscle ryanodine receptors. FEBS Lett.

[CR13] Geiger J, Zou AP, Campbell WB, Li PL (2000). Inhibition of cyclic ADP-ribose formation produces vasodilation in bovine coronary arteries. Hypertension.

[CR14] Graeff RM, Walseth TF, Hill HK, Lee HC (1996). Fluorescent analogs of cyclic ADP-ribose: synthesis, spectral characterization, and use. Biochemistry.

[CR15] Grimm W, Maisch B (2002). Sudden cardiac death in dilated cardiomyopathy—therapeutic options. Herz.

[CR16] Gul R, Kim SY, Park KH, Kim BJ, Kim SJ, Im MJ, Kim UH (2008). A novel signaling pathway of ADP-ribosyl cyclase activation by angiotensin II in adult rat cardiomyocytes. Am J Physiol Heart Circ Physiol.

[CR17] Gul R, Park JH, Kim SY, Jang KY, Chae JK, Ko JK, Kim UH (2009). Inhibition of ADP-ribosyl cyclase attenuates angiotensin II-induced cardiac hypertrophy. Cardiovasc Res.

[CR18] Guo X, Laflamme MA, Becker PL (1996). Cyclic ADP-ribose does not regulate sarcoplasmic reticulum Ca^2+^ release in intact cardiac myocytes. Circ Res.

[CR19] Gwathmey JK, Slawsky MT, Hajjar RJ, Briggs GM, Morgan JP (1990). Role of intracellular calcium handling in force–interval relationships of human ventricular myocardium. J Clin Invest.

[CR20] Hellmich MR, Strumwasser F (1991). Purification and characterization of a molluscan egg-specific NADase, a second-messenger enzyme. Cell Regul.

[CR21] Higashida H, Zhang JS, Hashii M, Shintaku M, Higashida C, Takeda Y (2000). Angiotensin II stimulates cyclic ADP ribose formation in neonatal rat cardiac myocytes. Biochem J.

[CR22] Hiraoka M (1987). Characteristics of triggered-activity and delayed afterdepolarization in responses to the electrical stimulation. Jpn Circ J.

[CR23] Hirata Y, Kimura N, Sato K, Ohsugi Y, Takasawa S, Okamoto H (1994). ADP ribosyl cyclase activity of a novel bone marrow stromal cell surface molecule, BST-1. FEBS Lett.

[CR24] Hochuli E, Bannwarth W, Döbeli H, Gentz R, Stüber D (1988). Genetic approach to facilitate purification of recombinant proteins with a novel metal chelate adsorbent. Nat Biotechnol.

[CR25] Hoffman BF, Rosen MR (1981). Cellular mechanisms for cardiac arrhythmias. Circ Res.

[CR26] Howard M, Grimaldi JC, Bazan JF, Lund FE, Santos-Argumedo L, Parkhouse RM, Walseth TF, Lee HC (1993) Formation and hydrolysis of cyclic ADP-ribose catalyzed by lymphocyte antigen CD38. Science 262:1056–1059 10.1126/science.8235624 8235624

[CR27] Iino S, Cui Y, Galione A, Terrar DA (1997). Actions of cADP-ribose and its antagonists on contraction in guinea pig isolated ventricular myocytes. Circ Res.

[CR28] Itoh M, Ishihara K, Tomizawa H, Tanaka H, Kobune Y, Ishikawa J (1994). Molecular cloning of murine BST-1 having homology with CD38 and *Aplysia* ADP-ribosyl cyclase. Biochem Biophys Res Commun.

[CR29] Kim SY, Gul R, Rah SY, Kim SH, Park SK, Im MJ (2008). Molecular mechanism of ADP-ribosyl cyclase activation in angiotensin II signaling in murine mesangial cells. Am J Physiol Renal Physiol.

[CR30] Kitts PA, Possee RD (1993). A method for producing recombinant baculovirus expression vectors at high frequency. Biotechniques.

[CR31] Kranias EG, Schwartz A, Jungmann RA (1982). Characterization of cyclic 3′:5′-amp-dependent protein kinase in sarcoplasmic reticulum and cytosol of canine myocardium. Biochim Biophys Acta.

[CR32] Kushnir A, Marks AR (2010). The ryanodine receptor in cardiac physiology and disease. Adv Pharmacol.

[CR33] Lee HC, Walseth TF, Bratt GT, Hayes RN, Clapper DL (1989). Structural determination of a cyclic metabolite of NAD^+^ with intracellular Ca^2+^ mobilizing activity. J Biol Chem.

[CR34] Lee HC, Aarhus R (1991). ADP-ribosyl cyclase: an enzyme that cyclizes NAD^+^ into a calcium-mobilizing metabolite. Cell Regul.

[CR35] Lee HC (2000). Enzymatic functions and structures of CD38 and homologs. Chem Immunol.

[CR36] Lehnart SE, Mongillo M, Bellinger A, Lindegger N, Chen BX, Hsueh W (2008). Leaky Ca^2+^ release channel/ryanodine receptor 2 causes seizures and sudden cardiac death in mice. J Clin Invest.

[CR37] Lehnart SE, Maier LS, Hasenfuss G (2009). Abnormalities of calcium metabolism and myocardial contractility depression in the failing heart. Heart Fail Rev.

[CR38] Malavasi F, Deaglio S, Ferrero E, Funaro A, Sancho J, Ausiello CM (2006). CD38 and CD157 as receptors of the immune system: a bridge between innate and adaptive immunity. Mol Med.

[CR39] Lukyanenko V, Györke I, Wiesner TF, Györke S (2001). Potentiation of Ca^2+^ release by cADP-ribose in the heart is mediated by enhanced SR Ca^2+^ uptake into the sarcoplasmic reticulum. Circ Res.

[CR40] Meszaros LG, Bak J, Chu A (1993). Cyclic ADP-ribose as an endogenous regulator of the non-skeletal type ryanodine receptor Ca^2+^ channel. Nature.

[CR41] Meszaros LG, Wrenn RW, Varadi G (1997). Sarcoplasmic reticulum-associated and protein kinase C-regulated ADP-ribosyl cyclase in cardiac muscle. Biochem Biophys Res Comm.

[CR42] Nam TS, Choi SH, Rah SY, Kim SY, Kim WJ, Im MJ (2006). Discovery of a small-molecule inhibitor of kidney ADP-ribosyl cyclase: implication for intracellular calcium signal mediated by cyclic ADP-ribose. Exp Mol Med.

[CR43] Prakash YS, Kannan MS, Walseth TF, Sieck GC (2000). cADP ribose and [Ca^2+^]_i_ in rat cardiac myocytes. AJP-Heart.

[CR44] Rakovic S, Galione A, Ashamu GA, Potter BVL, Terrar DA (1996). A specific cyclic ADP-ribose antagonist inhibits cardiac excitation–contraction coupling. Curr Biol.

[CR45] Rakovic S, Cui Y, Iino S, Galione A, Ashamu GA, Potter BVL, Terrar DA (1999). An antagonist of cADP-ribose inhibits arrhythmogenic oscillations of intracellular Ca^2+^ in heart cells. J Biol Chem.

[CR46] Rakovic S, Terrar DA, Lee HC (2000). Calcium signaling by cADPR in cardiac myocytes. Cyclic ADP-ribose and NAADP. Structures, metabolism and functions.

[CR47] Sitsapesan R, McGarry SJ, Williams AJ (1994). Cyclic ADP-ribose competes with ATP for the adenine nucleotide binding site on the cardiac ryanodine receptor Ca^2+^ release channel. Circ Res.

[CR48] States DJ, Walseth DF, Lee HC (1992). Similarities in amino acid sequences of *Aplysia* ADP-ribosyl cyclase and human lymphocyte antigen CD38. Trends Biochem Sci.

[CR49] Stewart R, Song L, Carter SM, Sigalas C, Zaccai NR, Kanamarlapudi V (2008). Single-channel characterization of the rabbit recombinant RyR2 reveals a novel inactivation property of physiological concentrations of ATP. J Membr Biol.

[CR50] Tang WX, Chen YF, Zou AP, Campbell WB, Li PL (2002). Role of FKBP12.6 in cADPR-induced activation of reconstituted ryanodine receptors from arterial smooth muscle cells. AJP-heart.

[CR51] Takasawa S, Nata K, Yonekura H, Okamoto H (1993). Cyclic ADP-ribose in insulin secretion from pancreatic β-cells. Science.

[CR52] Vaughn JL, Goodwin RH, Tompkins GJ, McCawley P (1977). The establishment of two cell lines from the insect *Spodoptera frugiperda* (Lepidoptera; Noctuidae). In Vitro.

[CR53] Venturi E, Pitt S, Galfre E, Sitsapesan R (2010) From eggs to hearts: what is the link between cyclic ADP-ribose and ryanodine receptors? Cardiovasc Ther. doi:10.1111/j.1755-5922.2010.00236.x 10.1111/j.1755-5922.2010.00236.x 21176119

[CR54] Xiao J, Tian X, Jones PP, Bolstad J, Kong H, Wang R (2007). Removal of FKBP12.6 does not alter the conductance and activation of the cardiac ryanodine receptor or the susceptibility to stress-induced ventricular arrhythmias. J Biol Chem.

[CR55] Xie GH, Rah SY, Yi KS, Han MK, Chae SW, Im MJ, Kim UH (2003). Increase of intracellular Ca^2+^ during ischemia/reperfusion injury of heart is mediated by cyclic ADP-ribose. Biochem Biophys Res Commun.

[CR56] Xie GH, Rah SY, Kim SJ, Nam TS, Ha KC, Chae SW (2005). ADP-ribosyl cyclase couples to cAMP signaling in the cardiomyocytes. Biochem Biophys Res Commun.

[CR57] Young GS, Choleris E, Lund FE, Kirkland JB (2006). Decreased cADPR and increased NAD + in the Cd38−/− mouse. Biochem Biophys Res Commun.

[CR58] Zhang X, Tallini YN, Chen Z, Gan L, Wei B, Doran R (2009). Dissociation of FKBP12.6 from ryanodine receptor type 2 is regulated by cyclic ADP-ribose but not beta-adrenergic stimulation in mouse cardiomyocytes. Cardiovasc Res.

